# From village to lab: An African scientist’s quest for a sustainable future

**DOI:** 10.1016/j.isci.2024.108936

**Published:** 2024-02-15

**Authors:** Steve Eshiemogie

**Affiliations:** 1 PhD Student, Chemical and Biological Engineering, Rensselaer Polytechnic Institute, New York, Troy 12180, USA

## Abstract

RBSA Runner-Up.

**Figure:**
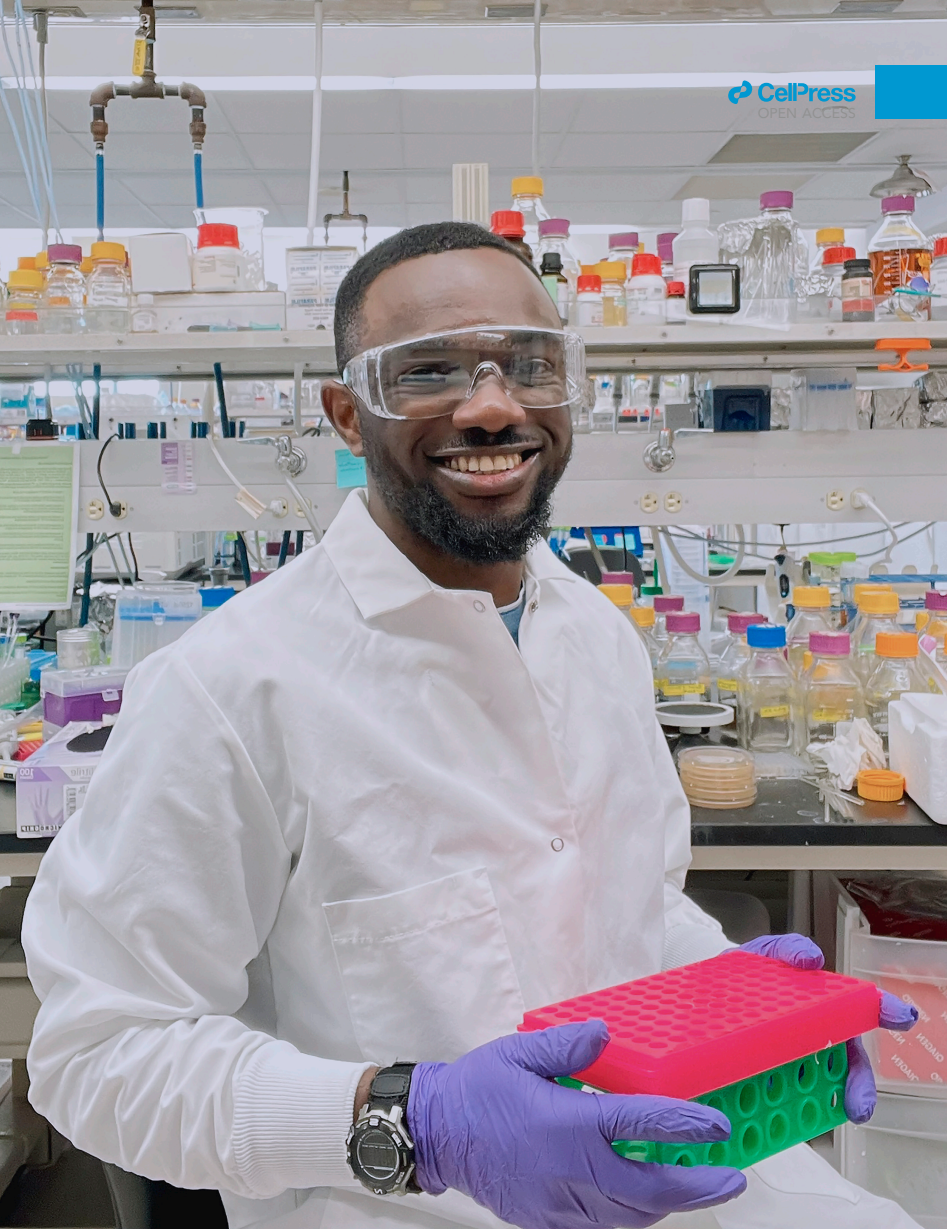
Above image: Steve Eshiemogie.

## Main text

Africa is often portrayed by the global media as a “rural village” with starving, pot-bellied out-of-school children roaming the streets and struggling for survival. Many African elites frequently complain about these media stereotypes, emphasizing that not all of Africa is as underdeveloped as depicted by the media. I often find myself “grinning” at such rebuttals because I, personally, grew up in the very “rural village Africa” that the media portrays—the Africa with starving, out-of-school children, lacking access to clean water and electricity. I grew up in *Gonin-Gora*, a remote village in Kaduna state, Northern Nigeria, and I have vivid memories of my upbringing. I distinctly recall my childhood in *Gonin-Gora*, a region with a multitude of challenges that had become so ingrained in our lives that many of them ceased to be perceived as problems—we simply grew accustomed to them. Growing up, my community faced severe issues, such as recurrent terror attacks from religious extremist groups, prolonged electricity blackouts spanning months, and even years, as well as a lack of access to clean drinking water. Surprisingly, or better put, “miraculously”, we endured all these hardships. Many times, my school was forced to close due to terrorist attacks, compelling us to stay at home and hope for better days.

As a young boy at the time, these experiences left me deeply frightened and significantly influenced my personality and thought process to this day. Year after year, my classmates dropped out of school in increasing numbers. The male students left to focus on small-scale farming, while the females were married off to older men. I, however, aspired more for myself. I yearned for an education. I believed that attending school was my path to being part of those who could one day find solutions to the numerous challenges afflicting my community and my continent at large. I held the belief that someone had to change the status quo, someone had to address the energy crisis, the water crisis, malnutrition, subpar education, and poor healthcare facilities. It had to be someone. As a matter of fact, I have to be someone!

For the most part of my childhood, electricity was a luxury I did not have. I only truly experienced its benefits during occasional holidays spent with my uncle in Lagos, Nigeria. Upon returning from one such visit, I found myself inspired to find a way to locally generate electricity to power my house. This motivation led me to dive into the world of physics and chemistry, poring over textbooks to understand the fundamental principles of electricity generation. Intriguingly, I came across topics that discussed generating electric charges from unexpected sources like lemon fruits, or by rubbing a pen against one’s hair. These concepts held a unique allure for me, but what truly ignited my passion was the concept of harnessing energy from bacteria—a process known as anaerobic digestion. Learning about this concept marked the beginning of my fervent interest in science, specifically biotechnology, and fueled my determination to dedicate my life toward applying the principles of biotechnology for the sustainable growth and development of the African continent.

During my undergraduate program, my passion for biotechnology led me to become an undergraduate research fellow in a U.K. government-funded project called “ACTUATE”, which received the Global Challenges Research Fund. This project focused on biomethane production from waste biomass using bacteria cultures. Through my participation in this research, I gained valuable insights into the possibilities of engineering cellular metabolic pathways for the biosynthesis of high-value compounds like biopharmaceuticals and biofuels. Driven by my dedication to translational research even at undergraduate level, I founded two organizations. The first, Green Africa Initiative, a non-profit focused on educating young African students on alternative energy, environmental sustainability, and effective leadership for a sustainable future. The second, a startup named Greenergy, focused on the bioproduction of biofuels from biowaste materials. As the founder of Greenergy, my passion for developing innovative solutions for sustainable bioproduct synthesis motivated me to pursue a PhD in metabolic engineering.

Now as a Doctoral student at Rensselaer in New York, my research is focused on the metabolic engineering of bacteria to produce high-value products. While pursuing my graduate career in the US, I am still very well aware that millions of young African students in my homeland continue to face underprivileged circumstances. In response, I established the Steve-Eshiemogie scholarship, which provides full tuition support to high achieving students in the field of biological sciences. As I progress in my academic and professional career, I remain resolute in my commitment to bridging the gap between African students and the abundant opportunities in the field of science. My mission—as an African village scientist, is to empower the next generation of African scholars and foster a brighter, more inclusive future in the realm of scientific exploration and innovation.

